# Introduction of a mass burn casualty triage system in a hospital during a powder explosion disaster: a retrospective cohort study

**DOI:** 10.1186/s13017-018-0199-9

**Published:** 2018-08-29

**Authors:** Chip-Jin Ng, Shih-Hao You, I-Lin Wu, Yi-Ming Weng, Chung-Hsien Chaou, Cheng-Yu Chien, Chen-June Seak

**Affiliations:** 1 Department of Emergency Medicine, Chang Gung Memorial Hospital and Chang Gung University, Linko, Taiwan; 2 Department of Emergency, Taoyuan Armed Forces General Hospital, Taoyuan, Taiwan; 3 Department of Emergency Medicine, Prehospital Care Division, Taoyuan General Hospital, Ministry of Health and Welfare, No. 1492 Zhongshan Rd., Taoyuan Dist, Taoyuan City, 330 Taiwan; 4 Faculty of Medicine, National Yang-Ming University School of Medicine, Taipei, Taiwan; 5 Department of Emergency Medicine, Ton-Yen General Hospital, Zhubei, Hsinchu County Taiwan

**Keywords:** Triage, Mass casualty incidents, Burns, Explosions, Outcome assessment

## Abstract

**Background:**

The triage system used during an actual mass burn casualty (MBC) incident is a major focus of concern. This study introduces a MBC triage system that was used by a burn center during an actual MBC incident following a powder explosion in New Taipei City, Taiwan.

**Methods:**

This study retrospectively analyzed data from patients who were sent to the study hospital during a MBC incident. The patient list was retrieved from a national online management system. A MBC triage system was developed at the study hospital using the following modifiers: consciousness, breathing, and burn size. Medical records were retrieved from electronic records for analysis. Patient outcomes consisted of emergency department (ED) disposition and intervention.

**Results:**

The patient population was predominantly female (56.3%), with an average age of 24.9 years. Mean burn sizes relative to the TBSA of triage level I, II, and III patients were 57.9%, 40.5%, and 8.7%, respectively. ICU length of stay differed markedly according to triage level (mean days for levels I vs II vs III: 57.9 vs 39.9 vs 2.5 days; *p* < 0.001). Triage system levels I and II indicate ICU admission with a sensitivity of 93.9% (95%CI 80.4–98.3%) and a specificity of 86.7% (62.1–96.3%).

Overall, 3 (6.3%) patients were under-triaged. Two (4.2%) patients were over-triaged. Sixteen (48.5%) and 21 (63.6%) patients of triage levels I and II received endotracheal intubation and central venous catheterization, respectively. Sorting of the study population with simple triage and rapid treatment (START) showed great sensitivity (100.0%) but poor specificity (53.3%). The Taiwan Triage and Acuity Scale (TTAS) presented 87.9% sensitivity and 93.9% specificity.

**Conclusions:**

The current MBC triage algorithm served as a good indicator of ED disposition but might have raised excessive immediate attention and had the potential to exhaust the available resources. These findings add to our knowledge of the MBC triage system and should help future researchers in adjusting the triage criteria to fit actual disasters.

## Background

Mass casualty triage helps first responders to sort victims during a mass casualty incident [1, 2]. The priority of care according to the triage scale indicates response time, transportation, resource allocation, and patient treatment [3, 4]. “Simple triage and rapid treatment” (START), which was developed in the 1980s, is a widely used method [1, 5]. A responder first identifies walking wounded patients (green), followed by sorting patients into immediate (red) and delay (yellow) based on respiratory rate, perfusion, and mental status. A previous study, which evaluated the performance of START via a retrospective review, showed poor agreement between triage levels and outcomes [6–8]. There were concerns about the insufficiency of using START triage in mass burn casualty (MBC) incidents [2]. A universal triage system used in a mass burn casualty (MBC) incident during a gas leak or powder explosion has not been validated [9]. In addition to inhalation injuries, the burn matrix uses age and burn size to classify patients in such circumstances [4, 10, 11]. However, it might not be feasible to rapidly determine a precise burn size when confronted with numerous patients [12]. Nevertheless, the burn matrix might be appropriate for use by a specialist to determine the tier of care 48 h following injury [10]. In short, the optimum approach to MBC triage remains unclear [5]. The development of a MBC triage system based on an actual event should be validated.

### Importance

The triage system used during an actual MBC incident is a major focus of concern. Examination of the relationship between triage level and patient outcome can improve the triage system to promote future disaster preparedness.

### Goals of this investigation

This study introduces and validates the MBC triage system that was used by a burn center during an actual MBC incident following a powder explosion in New Taipei City, Taiwan.

## Methods

### Study design and setting

This study retrospectively analyzed data from patients who were sent to the study hospital during a MBC incident on June 27, 2015. The study was approved by the Chang Gung Memorial Hospital (CGMH) Institutional Review Board and was exempted from requiring informed consent and full committee review.

At 20:32 on June 27, 2015, a colored cornstarch powder explosion occurred during a party at the Formosa Fun Water Park in New Taipei City and injured 499 people. Of the injured, 75% suffered from second- to third-degree burns. The Ministry of Health and Welfare activated the regional Emergency Medical Operation Center and informed designated hospitals to prepare for the MBC incident. In total, 48 patients were sent to the study hospital. The first injured patient was registered at the emergency department (ED) at 21:21. Others were admitted over the following 4 h. The hospital activated its mass casualty incident response at 22:00 and ended the incident command system at 01:04 on June 28, 2015.

All triage nurses were trained in a departmental teaching program using the “Taiwan Triage and Acuity Scale” (TTAS). This included reading a manual and participating in workshops twice per year. All triage nurses had completed the program. Patients suffering from burn injuries were classified under the chief complaint category of burn injury (TTAS T1301) with the primary modifiers of respiratory distress (oxygen saturation), level of consciousness (Glasgow Coma Scale, GCS), hemodynamic status (blood pressure), and pain (a numeric pain rating scale). The secondary modifiers included the size of second- to third-degree burns relative to the total body surface area (TBSA). The detailed criteria of different TTAS levels for burn injury are as follows:

- TTAS level 1 for burn injury: severe respiratory distress (oxygen saturation < 90%), hemodynamic collapse, or coma (GCS 3–8)
- TTAS level 2 for burn injury: moderate respiratory distress (oxygen saturation < 92%), hemodynamic instability, unclear consciousness (GCS 9–13), 2nd- and 3rd-degree burns with a size of > 25%, burn over face/genital area/hand/foot, or suspected inhalation injury.
- TTAS level 3 for burn injury: mild respiratory distress (oxygen saturation 92–94%), abnormal blood pressure measurement without signs of shock, pain scale 9–10, or 2nd- and 3rd-degree burns with a size of 5–25%.
- TTAS level 4 for burn injury: pain scale 4–7, or 2nd- and 3rd-degree burns with a size of < 5%.
- TTAS level 5 for burn injury: pain scale < 4.

**Fig. 1:**
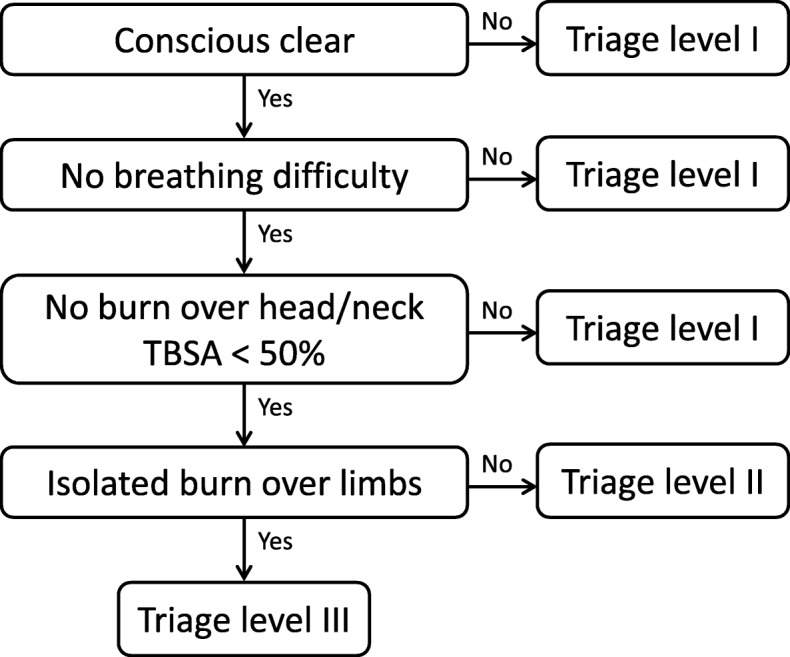
A mass burn casualty triage system was developed at the study hospital during this incident

However, there was no specific triage system for a MBC incident at the study hospital. Therefore, a MBC triage system, based on the consensus of triage nurses, was developed at the study hospital during this incident (Fig. 1). The triage nurses immediately sorted the patients after a primary survey during the mass casualty incident response. Patients were triaged as level I (emergency), II (urgent), or III (less urgent). Patients who presented with an altered level of consciousness or with any breathing difficulty were considered level I. Patients were then classified according to the individual burn size and body part involved. A burn size of TBSA > 50% or a burn injury over the head and neck area was classified as level I. Instead of a cutoff value of a burn size of > 25%, a TBSA > 50% was considered easier to identify and would garner higher levels of agreement among staff. Patients suffering from a burn injury that involved the torso area with TBSA < 50% were classified as level II. Those who presented with isolated limb burn injuries were classified as level III. In contrast to those with isolated limb burn injuries, patients with torso burn injuries might need a bed for further treatment and wound dressing changes.

Patients were then sent to red, yellow, and green zones for treatment according to triage levels I, II, and III, respectively. Patients received endotracheal intubation, central venous line access, and wound care from ED personnel according to their individual conditions. Patients were then either admitted to the intensive care unit (ICU)/ward or discharged, according to physicians’ assessments.

### Data collection and processing

Data were collected using several methods. First, on-duty emergency medical technicians registered patients who were affected by the MBC incident. The patient list, designated hospital, and time of registration were abstracted via an online emergency medical management system. Second, the patient list was then confirmed with the ED boarding system of the study hospital. All patients sent to CGMH from the MBC incident at the Formosa Fun Water Park explosion on June 27, 2015, were enrolled. Those who were transferred from other hospitals were excluded. Third, we reviewed the medical records and collected data using a standardized reporting template with clear definitions and codes. Data were retrieved from electronic records.

Background demographic characteristics of the enrolled patients, including age, gender, triage level, 2nd- or 3rd-degree burn size relative to the TBSA, escharotomy or tangential excision of necrotic tissue, number of procedures, abbreviated burn severity index (ABSI), and injury severity score (ISS), were abstracted. Data on ED interventions with endotracheal intubation and/or central venous catheterization (CVC) were recorded. Patient outcomes, including ED disposition (admitted to ICU/ward, discharged), ICU/ward length of stay (LOS), and mortality, were collected.

To compare the performance of START, TTAS, and the MBC triage used in the study hospital, the study population was assigned to different START/TTAS levels according to the records. The sensitivity and specificity of START and TTAS triage to predict ICU admission were calculated. The TTAS criteria for burn injury are listed above. The study population was retrospectively assigned into different START triage categories based on certain assumptions and the collected data as follows: START triage category immediate (red): unclear consciousness (GCS < 14), respiratory rate > 30, radial pulse absent or mean arterial pressure less than 65 mmHg and START triage category minor (green): patients allocated to the treatment area without beds.

All others patients were assigned to “delay (yellow) category”.

### Outcome measures

The primary outcome measure was ED disposition, including the proportion of ICU/ward admissions. Secondary outcome measures included ED interventions (i.e., endotracheal intubation, CVC, ICU/hospital LOS, and mortality).

### Primary data analysis

Data were analyzed using SPSS software (ver. 13.0 for Windows; SPSS Inc., Chicago, IL, USA). In the descriptive analysis, normality tests for continuous variables were performed. Means and standard deviations were used to describe the central tendency and spread of continuous variables. Categorical variables are presented as counts and percentages and were compared using the chi-squared test or Fisher’s exact test. Comparisons between groups in terms of the continuous variables were performed using analysis of variance (ANOVA) as appropriate. A trend test was also used to examine the sequential effect of the triage levels. A Kaplan–Meier survival curve was used to describe relationships between the length-of-stay curves of patients at different triage levels. A log-rank test was used to confirm differences in survival curves. A *p* value < 0.05 was considered to indicate statistical significance.

## Results

Data from 48 patients were analyzed, and the patients’ demographic characteristics are presented in Table 1. The study population was predominantly female (56.3%), with an average age of 24.9 years. The mean of 2nd- and 3rd-degree burn sizes relative to the TBSA of triage level I, II, and III patients were 57.9%, 40.5%, and 8.7%, respectively. The outcomes of different triage levels were compared. There was a trend toward a difference between groups at different triage levels with regard to ICU admission (level I vs II vs III, 100.0 vs 89.5 vs 20.0%; *p* < 0.001). There was a significant difference in ED intervention between the groups (level I vs II vs III: endotracheal intubation, 92.9 vs 15.8 vs 6.7%, *p* < 0.001; CVC, 71.4 vs 57.9 vs 6.7%, *p* < 0.001). Overall, 3 (9.1%) patients, who were admitted to the ICU with initial triage level III, were under-triaged. Two patients were admitted to the ICU at the ED, and one was admitted to the ICU via a ward. The first patient, who experienced a 20% TBSA burn injury, had full consciousness and normal breathing and was without head or neck burn injury. This patient was triaged as level III at the ED and suffered from shortness of breath during ED boarding 6 h after the accident. The patient received nasal intubation and was then admitted to the ICU. The second and third patients, who experienced 24 and 18% TBSA burn injuries, respectively, were admitted to the ICU. These two patients were admitted due to difficult wound care according to a plastic surgeon’s judgment.

**Table 1:** Patient characteristics and outcomes comparison between triage levels

	Overall		Triage level I		Triage level II		Triage level III		*p* value for
	*N* = 48		*N* = 14		*N* = 19		*N* = 15		Difference
Gender, *N* (%)									0.623
Female	27	(56.3)	9	(64.3)	11	(57.9)	7	(46.7)	
Male	21	(43.7)	5	(35.7)	8	(72.7)	8	(53.3)	
Age in years, mean (SD)	24.9	(8.8)	26.6	(11.6)	25	(9.0)	23.1	(3.5)	0.588
TBSA of 2nd to 3rd-degree of burn (%), mean (SD)	35.6	(23.8)	57.9	(15.4)	40.5	(15.9)	8.7	(7.8)	< 0.001
ED intervention, *N* (%)									
Endotracheal intubation	17	(35.4)	13	(92.9)	3	(15.8)	1	(6.7)	< 0.001
CVC	22	(45.8)	10	(71.4)	11	(57.9)	1	(6.7)	< 0.001
Surgery, *N* (%)									< 0.001
Escharotomy	22	(45.8)	12	(85.7)	9	(47.4)	1	(6.7)	
Tangential excision of necrotic skin	17	(35.4)	2	(14.3)	10	(52.6)	5	(33.3)	
No surgery	9	(18.8)	0	(0)	0	(0)	9	(60.0)	
Overall procedures, *N* (%)	4.3	(3.6)	6.2	(2.3)	5.3	(3.9)	1.0	(1.4)	< 0.001
ED disposition, *N* (%)									< 0.001
ICU	34	(70.8)	14	(100)	17	(89.5)	3	(20.0)	
Ward	5	(10.4)	0	(0)	2	(10.5)	3	(20.0)	
Discharge	8	(16.7)	0	(0)	0	(0)	8	(53.3)	
Transfer	1	(2.1)	0	(0)	0	(0)	1	(6.7)	
ABSI, mean (SD)	7.3	(2.9)	10.3	(1.6)	7.6	(1.7)	4.1	(1.1)	< 0.001
ISS, mean (SD)	16.5	(13.2)	27.3	(14.1)	19.4	(6.8)	2.7	(2.8)	< 0.001
LOS in days, mean (SD)									
ICU	32.2	(30.9)	57.9	(16.6)	39.9	(30.9)	2.5	(6.6)	< 0.001
Hospital	48.6	(37.0)	73.9	(15.3)	62.2	(36.4)	12.0	(17.1)	< 0.001
Mortality, *N* (%)	2	(4.2)	2	(14.3)	0	(0)	0	(0)	0.081

*Abbreviations: SD* standard deviation, *TBSA* total body surface area, *ICU* intensive care unit, *ED* emergency department, *CVC* central venous catheterization, *ABSI* abbreviated burn severity index, *ISS* injury severity score, *LOS* length of stay

In contrast, 2 (6.1%) patients were over-triaged and were classified as levels I and II without mortality or ICU admission. Only one of 15 (6.7%) patients at triage level III received endotracheal intubation and CVC in the ED, whereas 16 (48.5%) and 21 (63.6%) patients at triage levels I and II received endotracheal intubation and CVC, respectively. A total of 22 (45.8%) patients received escharotomy. Table 2 demonstrates the performance of the triage system regarding the prediction of ICU admission, endotracheal intubation, and CVC at the ED. The overall MBC triage system, which indicated levels I and II vs level III, predicted ICU admission with a sensitivity of 93.9% (95%CI 80.4–98.3%) and a specificity of 86.7% (62.1–96.3%).

**Table 2:** The performance of the MBC triage system in the study

	ICU admission (%), 95%CI	ET Intubation (%), 95%CI	CVC (%), 95%CI	Escharotomy (%), 95%CI
Triage category I				
Sensitivity	42.4 (27.2–59.2)	76.5 (52.7–90.4)	45.5 (26.9–65.3)	54.6 (34.7–73.1)
Specificity	100.0 (79.6–100.0)	96.8 (83.8–99.4)	84.6 (66.5–93.3)	92.3 (75.9–97.9)
Triage category II				
Sensitivity	51.5 (35.2–67.5)	17.7 (6.2–41.0)	50.0 (30.7–69.3)	40.9 (23.3–61.3)
Specificity	86.7 (62.1–96.3)	48.4 (32.0–65.2)	69.2 (50.0–83.5)	61.5 (42.5–77.6)
Triage category III				
Sensitivity	6.1 (1.7–19.6)	5.9 (1.1–27.0)	4.6 (8.0–21.8)	4.6 (8–21.8)
Specificity	13.3 (3.7–37.9)	54.8 (37.8–70.8)	46.2 (28.8–64.5)	46.2 (28.8–64.5)
Triage category MBC*				
Sensitivity	93.9 (80.4–98.3)	94.1 (73.0–99.0)	95.5 (78.2–99.2)	95.5 (78.2–99.2)
Specificity	86.7 (62.1–96.3)	45.2 (29.2–62.2)	53.9 (35.5–71.2)	53.9 (35.5–71.2)

*Abbreviations: MBC* mass burn casualty, *ICU* intensive care unit, *CI* confidence interval, *ET* endotracheal tube, *CVC* central venous catheterization

*Triage category MBC indicates the triage categories I and II vs III in predicting ICU admission and interventions

**Fig. 2:**
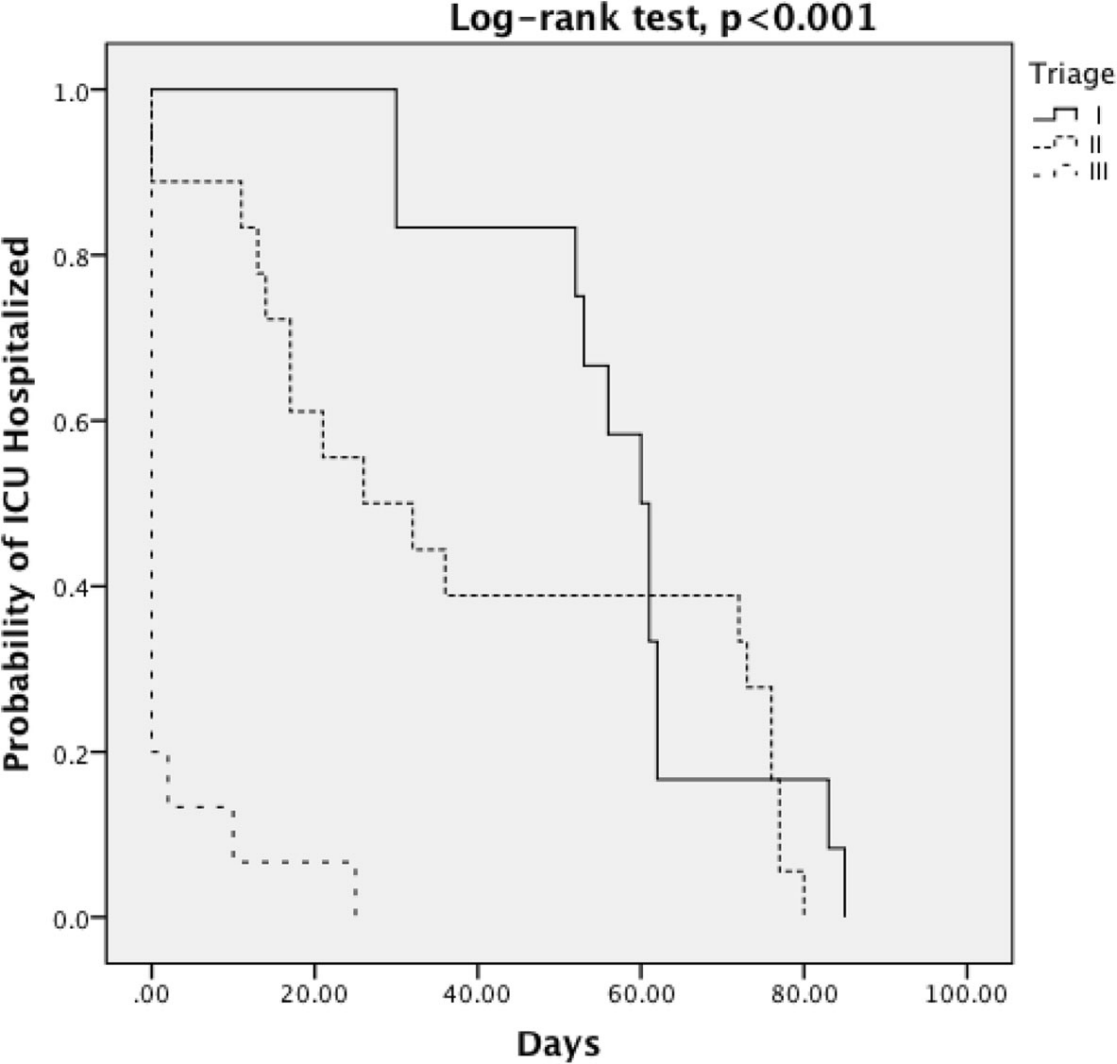
A Kaplan–Meier survival curve was used to describe relationships between the length-of-stay curves of patients at different triage levels

Both ICU and total hospital LOSs significantly differed between groups. Patients at triage level I had the longest ICU and hospital LOSs, with mean LOSs of 32.2 and 48.6 days, respectively. There was no mortality in the triage level II and III groups, and two deaths occurred in the level I patients. The percentage of time in the ICU according to the total number of hospitalization days in the different groups is plotted in Fig. 2 using a Kaplan–Meier estimate. The survival curves (probability of remaining in the ICU) differed markedly among the triage levels (*p* < 0.001, log-rank test).

Table 3 demonstrates the sensitivity and the specificity of START, TTAS, and the MBC triage categories in predicting ICU admission. While START had 100.0% sensitivity but poor specificity (53.3%), the MBC triage category showed comparable performance with TTAS. The predictability of ED intervention and escharotomy among different triage systems revealed similar findings (TTAS: endotracheal intubation sensitivity/specificity: 94.1%/54.8%; CVC: 90.9%/61.5%; escharotomy: 95.5%/65.4%; START: endotracheal intubation: 100.0%/28.6%; CVC: 100.0%/34.8%; escharotomy: 100.0%/33.3%).

**Table 3:** The comparison between different triage systems to predict the ICU admission

	Triage category MBC^*^	TTAS^#^	START^$^
Sensitivity (%), 95% CI	93.9 (80.4–98.3)	87.9 (72.7–95.2)	100.0 (87.1–100.0)
Specificity (%), 95% CI	86.7 (62.1–96.3)	93.3 (70.2–98.8)	53.3 (30.1–75.2)

*Abbreviations: ICU* intensive care unit, *MBC* mass burn casualty, *CI* confidence interval, *TTAS* Taiwan Triage Acuity Scale, *START* simple triage and rapid treatment

*Triage category MBC indicates the triage categories I and II vs III in predicting ICU admission

^#^TTAS levels 1 and 2 vs levels 3–5 in predicting ICU admission

^$^START triage red (immediate) and yellow (delayed) vs green (minor) in predicting ICU admission

## Discussion

Mass casualty incidents have the potential to overwhelm the medical resources of even highly developed nations; thus, a feasible triage strategy is important to ensure that resources are used effectively [1, 13]. Focusing on patient outcomes, this retrospective study examined a triage method used by a medical center during a colored cornstarch powder explosion MBC incident. Several aspects should be discussed in relation to the MBC triage system addressed in this study. During a MBC incident, it might not be practical to determine the triage level by inputting all the modifiers using a computer system or coding book according to the TTAS criteria. TTAS was developed and modified based on the Canada Triage and Acuity Scale (CTAS), which is widely used in Taiwan [14–16]. There was no specific triage method for such a MBC incident. Thus, a modified triage system based on the current method was developed. A MBC triage system developed in such a short period should be simple and easy to use without developing new measurement tools [6, 7]. Additionally, the criteria for different triage levels should be easy to identify and recall and result in as little inter-rater variation as possible. Testing for responsiveness is the universal first step when a first responder approaches a victim [17]. Patients with altered level of consciousness were triaged as level I (red); these patients may require immediate intervention. Second, breathing is assessed. A patient suffering from an inhalation injury might initially present with a normal breathing pattern. Thus, inhalation injuries should be evaluated in terms of any suspicious signs, such as facial burns, nose hair burns, and hoarseness. Once respiratory distress progresses, laryngeal edema might result in a difficult airway, possibly requiring surgical airway establishment [18]. As a result, the study triage protocol stipulated emergency evaluation and management for those who presented with any difficulty in breathing. Third, patients with full consciousness and normal breathing were further classified according to second- and third-degree burn size and body part. TTAS, a five-level triage scale [14], considers a TBSA of > 25% as a secondary modifier to classify patients as level II without age adjustment. More than 40% TBSA affected by second- and third-degree burns in patients aged 20–29.9 years was considered to present a medium-to-low survival rate according the American Burn Association benefit-to-resources grid, which indicates a severe form of injury [9, 10]. This study used a cutoff criterion of 50% TBSA to sort the study population in such a young age group. The current triage algorithm appeared to be a robust screening tool for ED implementation while facing a MBC incident.

Outcome assessment was performed for the enrolled patients. For the primary outcome of ICU admission, the results revealed an acceptable sensitivity (93.9%) but insufficient specificity (86.7%). There was an obvious trend toward a different length of hospital stay between triage levels. The ABSI and ISS also revealed a significant difference between triage levels, which indicated the survival and prognosis. One patient (2.1%) was intubated, and 3 (6.3%) were admitted to the ICU with an initial triage level of III. Approximately half of patients in triage level III were admitted for further care. Under-triage may endanger patient safety due to delayed treatment, promoting unfavorable outcomes. Therefore, it is not feasible to divert triage level III patients to other resources. Regarding over-triage, all patients but 2 (4.2%) of triage levels I and II were admitted to the ICU. Previous studies showed that prehospital triage had a high over-triage rate [6–8]. Kahn et al. conducted an outcome assessment after a true disaster to determine whether START triage levels match patients’ actual clinical status [6]. The outcome consisted of hospital admission and modified Baxt criteria, such as chest decompression and airway procedures. The report concluded that an acceptable under-triage but a substantial amount of over-triage had occurred. In another report, Hoff et al. also noted 23.3% over-triage and 5.8% under-triage by applying the START algorithm during a state triage tag exercise in 2011 [19]. Our study result echoes that of the previous study showing great sensitivity but very poor specificity of the START triage to sort the study population. Further analysis showed comparable performance of the MBC triage used in this study to that of TTAS but much better specificity than START. However, TTAS and START were applied retrospectively with 100% compliance using all available measurements at ED, which might not be available at the triage area. Therefore, overestimation of the performance of TTAS and START might have occurred. The current MBC triage system performed comparably to TTAS but better than START.

Regarding endotracheal intubation, triage level I identified that 76.4% (13/17) of patients required endotracheal intubation. In contrast, only 45.5% (10/22) of patients who received CVC had been classified as triage level I. Overall triage levels I and II identified approximately 95% patients who required emergent endotracheal intubation and CVC. A validated triage system should be consistent with patient outcomes, and the MBC triage tool used in this study might not precisely identify the immediate intervention needed at ED [20, 21]. Although the current MBC triage algorithm may contribute to ED disposition, resources may be exhausted due to over-triage for immediate needs.

### Limitations

This study should be interpreted in the context of the following limitations. First, due to its retrospective nature, small sample size, and specific age group of patients from a single medical center, selection bias may have affected the results. Second, there may have been unmeasured confounders, such as triage nurse experience, the triage criteria used for each patient, the influential factor of physician judgment, and ED disposition. Third, this study was conducted at a university-affiliated teaching hospital after a MBC incident, which might limit the generalizability of the findings. Additionally, the number of patients sent to the study hospital did not exceed the surge capacity of the hospital. A comparison of the validity of these study results with those from a different setting would be of interest. Fourth, the comparison among different triage systems might have been based on assumptions, including full compliance with the triage criteria. In addition, some data were gathered at the treatment area rather than during triage. Therefore, underestimation of the MBC triage system compared to START or TTAS might have occurred.

## Conclusions

The current MBC triage algorithm served as a superior indicator of ED disposition relative to START with a comparable performance to TTAS in a MBC incident. This triage system is validated with patient outcomes instead of immediate attention with the potential for resource depletion. The outcomes analysis of the triage system should extend beyond ED disposition and prognosis and encompass emergent intervention. These findings add to our knowledge of the MBC triage system and should help future research to adjust the triage criteria to fit actual disasters.

## Abbreviations

ABSI: Abbreviated burn severity index
CGMH: Chang Gung Memorial Hospital
CVC: Central venous catheterization
ED: Emergency department
ICU: Intensive care unit
LOS: length of stay
MBC: Mass burn casualty
START: Simple triage and rapid treatment
TBSA: Total body surface area
TTAS: Taiwan Triage and Acuity Scale
